# The Influence of Heat Treatment Temperature on Microstructures and Mechanical Properties of Titanium Alloy Fabricated by Laser Melting Deposition

**DOI:** 10.3390/ma13184087

**Published:** 2020-09-15

**Authors:** Wei Wang, Xiaowen Xu, Ruixin Ma, Guojian Xu, Weijun Liu, Fei Xing

**Affiliations:** 1 School of Mechanical Engineering, Shenyang University of Technology, Liaoning 110870, China; 17824947368@163.com (X.X.); siasky@163.com (F.X.); 2 School of Material Science and Engineering, Shenyang University of Technology, Liaoning 110870, China; ruixinma@163.com

**Keywords:** laser melting deposition, TC4 titanium alloy, normalizing temperature, microstructure, mechanical properties

## Abstract

Ti-6Al-4V (TC4) titanium alloy parts were successfully fabricated by laser melting deposition (LMD) technology in this study. Proper normalizing temperatures were presented in detailed for bulk LMD specimens. Optical microscope, scanning electron microscopy, X-ray diffraction, and electronic universal testing machine were used to characterize the microstructures, phase compositions, the tensile properties and hardness of the TC4 alloy parts treated using different normalizing temperature. The experimental results showed that the as-fabricated LMD specimens’ microstructures mainly consisted of α-Ti phase with a small amount of β-Ti phase. After normalizing treatment, in the area of α-Ti phase, the recrystallized length and width of α-Ti phase both increased. When normalizing in the (α + β) phase field, the elongated primary α-Ti phase in the as-deposited state was truncated due to the precipitation of β-Ti phase and became a short rod-like primary α-Ti phase. In as-fabricated microstructure, the β-Ti phase was precipitated between different short rod-shaped α-Ti phases distributed as basketweave. After normalizing treatment at 990 for two hours with subsequent air cooling, the TC4 titanium alloy had significant different microstructures from original sample produced by LMD. The normalizing treatment methods and temperature can be qualified as a prospective heat treatment of titanium alloy fabricating by laser melting deposition.

## 1. Introduction

Titanium alloys are widely used in aerospace and biomedical fields due to their high tensile strength and fatigue strength, low elastic modulus and density, high temperature performance, and good corrosion resistance [1,2,3]. As a medium strength α + β titanium alloy, Ti-6Al-4V is widely used in fields of aircrafts and aeroengines [4,5]. However, it is difficult to process due to the high melting point, high melting activity, and high deformation resistance of Ti-6Al-4V [6,7].

Additive manufacturing (AM), commonly known as 3D printing, makes physical objects from special metal materials, non-metallic materials and medical biological materials through software and numerical control system integrating computer-aided design, material processing and molding technology based on digital model files [8,9]. Laser melting deposition (LMD) is a rapid solidification forming technology based on layer-by-layer melting materials [10,11]. It aims to prepare dense near-net-shape metal components [12,13]. Compared with the traditional manufacturing process of titanium alloy, it has unique technical and economic advantages, especially suitable for manufacturing aerospace components that are made of titanium alloy and other heterogeneous materials [14,15]. At present, the comprehensive mechanical properties of titanium alloy produced by LMD reaches the same level as forging process. However, large residual stress of workpiece still resulted from rapid cooling and solidification limiting its application [16,17]. It is necessary to eliminate residual stress and further refine the microstructures to improve the mechanical properties of titanium alloy parts [18,19].

Heat treatment was widely conducted by many researchers to improve the mechanical properties of titanium alloy produced by LMD. Vrancken’s researches [20] about the annealing treatment of TC4 fabricated by SLM showed that the crystal growth was obviously fasterwhen the annealing temperature was higher than β transformation temperature, which resulted in transformation from β phase of columnar crystals to equiaxed crystals. Sercombe et al. [21] pointed out that the cooling method of laser melting depositonTi-6Al-7Nb heat treatment is closely related to the microstructure, and the microstructure after furnace cooling is larger than that after air cooling. Zhou Qingjun [22] presented that after double annealing of laser melt deposited TC11 at 950 °C for 1 h and 550 °C for 2 h, the continuous grain boundary α phase was almost completely broken. The result showed the mechanical anisotropy property at room temperature was eliminated completely and the plasticity was greatly reduced. Dai et al. [23] studied the effects of heat treatment temperature on the corrosion resistance of SLM-formed TC4 titanium alloy with poor creep resistances. Gerrit et al. [24] reported the phase fraction and tensile properties of TC4 titanium alloys parts with extensive heat treatment temperatures via a laser melting deposition process. Their results indicate that the heat treatment can eliminate the residual stress and improve its mechanical properties [25]. Therefore, heat treatment can effectively control the microstructure of TC4 alloy after DLM forming.

In recent years, many scholars have carried out detailed and extensive studies on the effects of annealing on the microstructure and properties of laser melting titanium alloys. However, little research focuses on the normalizing heat treatment process of titanium alloys. At the same time, after considering the efficiency and performance, the normalizing treatment is selected in this experiment. The microstructure and mechanical properties of the original titanium alloy prepared by laser melting deposition and different normalizing conditions are compared. The influence of different normalizing temperature on the microstructure and mechanical properties is studied in detail. The formation mechanisms of tensile fracture under different temperatures were explored to improve the laser melting deposition in the future.

## 2. Experimental

Laser melting deposition titanium alloys experiments were carried out on LDM 8060 system produced by RAYCHAM (Nanjing, China) as shown. The laser beam was generated by a 4 KW semiconductor laser. Powder was fed into the processing zone through an airborne powder feeder with a continuous four-way powder feeding coaxial powder nozzle. An additional protective inert argon gas stream is 8 L·min^−1^ 99.99% argon shielding gas is purged in the inert gas chamber (O_2_ < 50 ppm) with the oxygen content less than 50 × 10^−6^ of water. The laser melting deposition parameters are listed in Table 1. The single layer thickness of specimen is about 0.6 mm in the experiments. The specimens prepared by LMD are normalized treatment.

**Figure 1:**
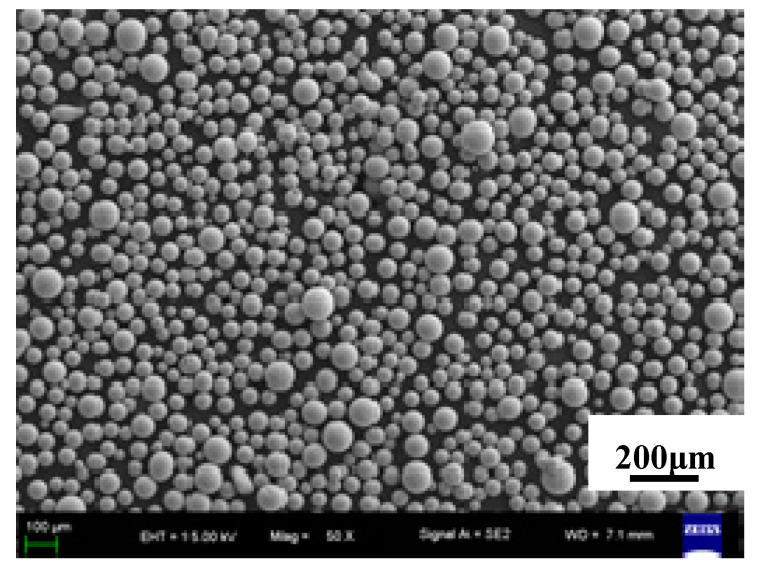
SEM photos of TC4 powder (500×).

Ti-6Al-4V for LMD was commercial materials with a particle size of 75–150 μm (Falcon Tech Co. Ltd., Suzhou, China) which was prepared with gas atomization technology as shown in Figure 1. The composition was shown in Table 2. Before the test, the powders were dried for 2 h at 150 °C in vacuum drying oven. The substrate was a piece of TC4 titanium alloy with a size of 150 × 150 × 20 mm^3^. The substrate surface was polished before LMD to remove the oxide film and surface defects on the surface, and then was cleaned by anhydrous ethanol and acetone to remove the impurities such as oil contamination. Samples 100 × 40 × 40 mm^3^ in size were built of powder by LMD with nominal composition.

Normalizing treatment was performed in a vacuum heat treatment furnace (KT-17500/JB-600, Zhengzhou, China). Table 3 listed the different heat treatment parameters applying to as-built laser melting deposition parts. Air cooling (AC) refers to cool down in air after the respective heat treatment cycle ends.

**Figure 2:**
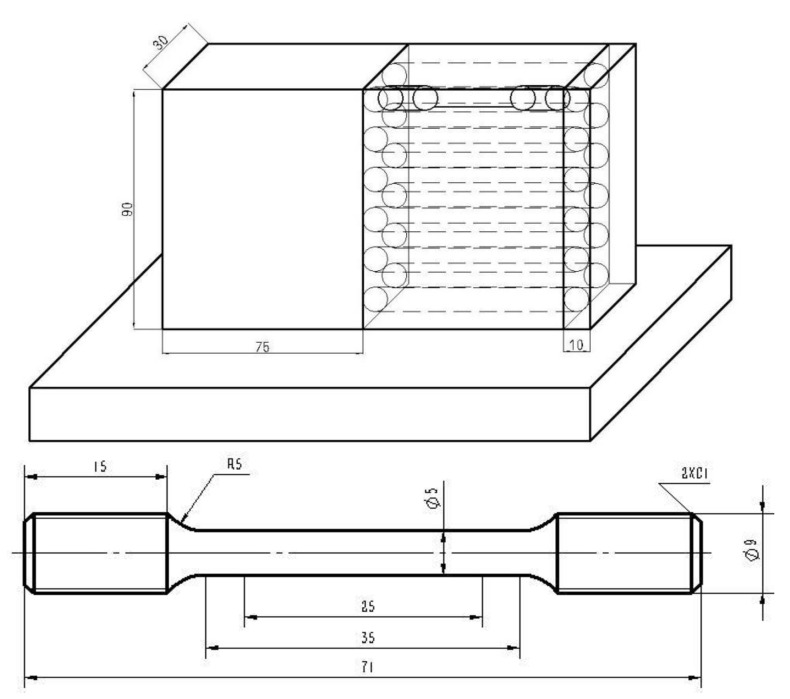
Schema of specimen wire cut electrical discharge machining (WEDM) in mm.

Metallographic specimens and room temperature tensile specimens were cut along the vertical laser scanning direction by wire cut electrical discharge machining (WEDM, Taizhou, China). They were prepared by standard mechanical grinding, polishing and etching in a mixed solution of 100 mL HF, 150 mL HNO_3_, and 500 mL H_2_O. The optical metallographic microscope (Carl Zeiss, ZX-10, Analytik Jena, Gina, Germany), field emission scanning electron microscope (Hitachi, SU8010, Tokyo, Japan) and scanning electron microscopy (Hitachi, S-4300, Tokyo, Japan) incorporating energy dispersive X-ray analysis were adopted to analyze the sample microstructures. A SHIMADZU X-ray diffraction (Cu Kα radiation, Tokyo, Japan) with a scanning speed of 8 °C·min^−1^ and a scanning range of 20~90 °C was used to detect the phase composition. The WDW-100 electronic universal testing machine (Jinan, China) was used to tensile test manufactured with a loading rate 2 mm·min^−1^. The length–width ratio and volume fraction of primary α-Ti phase were measured by Nano Measurer and Image-Pro Plus software. Tensile property of the specimens along longitudinal direction was evaluated on MTS880 test system (Eden Prairie, MN, USA) using cylindrical-like tensile specimen shown in Figure 2.

## 3. Results and Discussions

### 3.1. Microstructures

**Figure 3:**
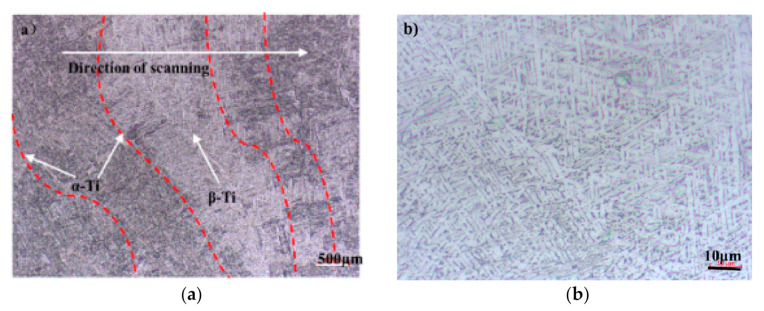
Macroscopic appearance of the as-deposited layer. (**a**) Bottom area, (**b**) top area.

The microstructure of the as-deposited layer near the fusion line is composed of epitaxial growth coarse columnar crystals. The columnar crystals growth along the direction of deposition which is basically perpendicular to the scanning direction of the laser beam is shown in Figure 3a. During the forming process of LDM, the temperature gradient near the fusion line is large and the crystal growth rate is slow resulted in small component supercooling, so columnar crystals are easy to form near the fusion line. At the top of the molten pool, the fast crystal growth rate resulted in large supercooling component due to the small temperature gradient. Accordingly, it is easy to form equiaxed crystals on the top of the molten pool [26]. This is because the small temperature gradient and the fast crystal growth rate with the increase of constitutional supercooling. If the thickness of equiaxed crystal growth zone at the top of molten pool is very thin, the remelting depth of the next layer is greater than the equiaxed crystal growth thickness shown in Figure 3b. Therefore, the equiaxed crystal growth zone will be melted out. The new as-deposited layer will continue to grow along the deposition direction on the basis of the columnar crystal of the previous as-deposited layer with the characteristics of directional solidification. The grain boundary of the primary β-Ti phase mainly occurs in the grain boundary of α-Ti phase. Nevertheless, the sub-structure of the original β-Ti phase is composed of the microstructure between the lath-like primary α-Ti phase.

**Figure 4:**
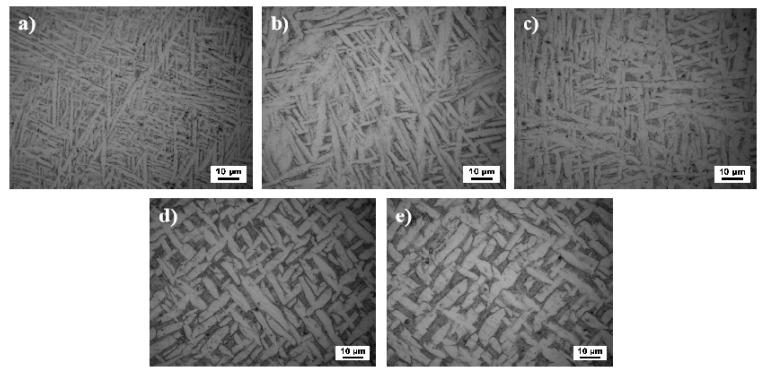
Normalizing microstructure at different temperatures. (**a**) As-deposited, (**b**) 810 °C/2 h/AC, (**c**) 870 °C/2 h/AC, (**d**) 930 °C/2 h/AC, (**e**) 990 °C/2 h/AC.

**Figure 5:**
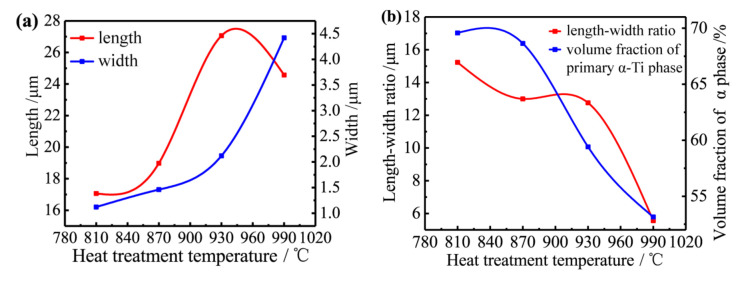
(**a**) Effect of normalizing temperature on the length and width of primary α-Ti phase, (**b**) effect of normalizing temperature on the aspect ratio and volume fraction of primary α-Ti phase.

**Figure 6:**
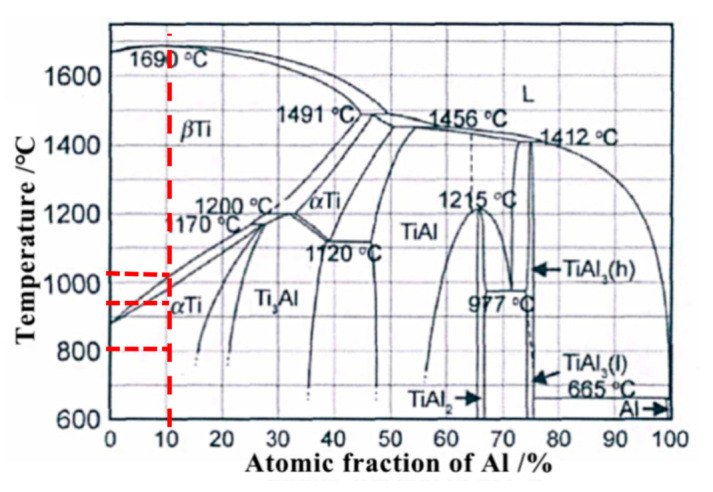
Ti-Al binary alloy phase diagram.

In the rapid heating melting and cooling solidification during the manufacturing process coarse columnar crystals with poor comprehensive properties are formed easily because of the molten pool introduced by the interaction of laser beam and powder flow. Additionally, the residual stress is inevitably produced resulting from non-holistic heating in the process. Therefore, normalized treatment is conducted on the LMD sample to improve the as-deposited microstructure and to eliminate the residual stress. The microstructures of the as-deposited and the normalized treatment specimens are shown in Figure 4. Figure 4a shows the microstructure of the as-deposited state. Figure 4b–e show the LMD samples normalized at the temperature of 810 °C, 870 °C, 930 °C, and 990 °C, respectively, heated for 2 h before air cooling. The microstructures of the as-deposited specimens are composed of a large number of elongated primary α-Ti phases and a small amount of β-Ti phases. At the normalizing temperature of 810 °C, the primary α-Ti phase is slightly coarser than the as-deposited phase, but the lath-like primary α-Ti phase is still uniform and fine. When the normalizing temperature reaches 870 °C, the coarsening ratio of primary α-Ti to the as-deposited state significantly increases along with the length and width direction. And it grows faster in the length direction. At the normalizing temperature of 930 °C, the coarsening of primary α-Ti phase is more obvious than that of the as-deposited phase, and the growth speed of length and width are almost same, so the ratio of length to width is basically unchanged as shown in Figure 4d. However, at the normalizing temperature of 930 °C, the α + β two-phase region exists. Due to the high heating temperature, the growth of primary α-Ti phase in the length direction is easy to intersect with other primary α-Ti and stop growing. It is to say that the truncation phenomenon occurs. So, the number of slender primary α-Ti phase decreases and gradually becomes a short rod-like shape. Meanwhile, the normalizing temperature in the two-phase region of α + β, the β-Ti phase begins to precipitate along the primary α-Ti laths leading to the decrease of the content of primary α-Ti phases as shown in Figure 5. Finally, when the normalizing temperature reaches 990 °C, as shown in Figure 4e, the primary α-Ti phase is cut off again and the short rod–like primary α-Ti phase transforms to the equiaxed crystal. It attributes that the phase is nearly liquid phase state from the diagram of Ti-Al binary alloy [27] in Figure 6 At the temperature of 990 °C, there is no primary α-Ti phase to hinder the migration of grain boundary which leads to the rapid migration of grain boundary. As a result, the grains of TC4 titanium alloy begin to grow rapidly nearby the transformation point of 990 °C. In addition, the primary α-Ti phase structure is relatively coarse after air cooling. This kind of α-Ti microstructure has high durability and creep resistance but low impact toughness. At the same time, the content of β-Ti phase increases and the content of primary α-Ti phase decreases compared with 930 °C. The microstructure between primary α-Ti phase (black gray color) is composed of two phases of α + β, which is verified by SEM and XRD analysis [28,29]. The microstructure is uniform in the shape of the basket-weave microstructures with the increase of normalizing temperature. Residual amount of β-Ti phase will increase at room temperature confirmed by XRD analysis later in this paper.

The influence of normalizing temperature on the length, width, length–width ratio and the aspect ratio and volume fraction of primary α-Ti phase are shown in Figure 5. It can be seen from Figure 5a that with the normalizing temperature increasing, the length of the lath-shaped microstructure first increases and then decreases, and the maximum value appears at the normalizing temperature of around 945 °C. As the increase of the normalizing temperature occurs, the width of the lath-shaped microstructure increases. Accordingly, growth rate becomes slower when the temperature is below 930 °C while becomes faster above 930 °C. Figure 5b showed that the length–width ratio of the lath-shaped microstructure decreases with the increase of temperature from 870 °C to 930 °C. What is more, the content of the primary α-Ti phase decreases gradually with the increase of temperature which becomes slower in the temperature range of 810~870 °C. After the temperature reaching 870 °C, it is preliminarily concluded that the heating temperature is close to the α+β two-phase region.

**Figure 7:**
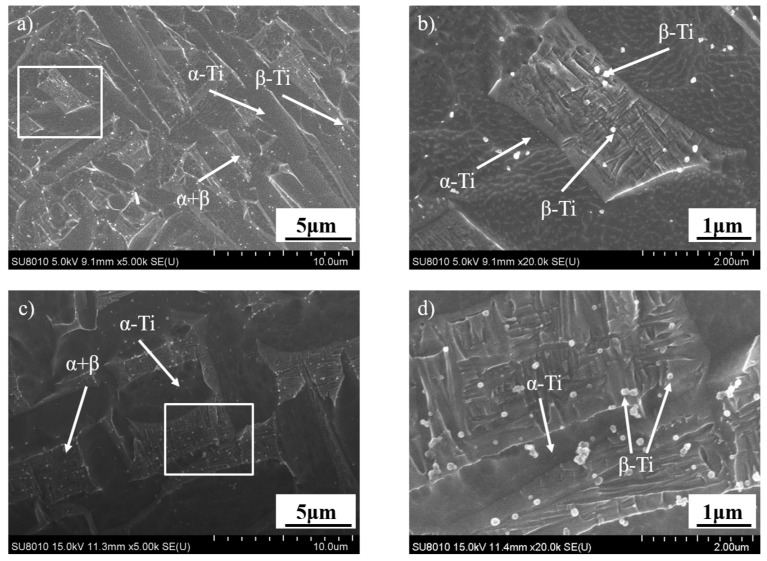
SEM images of normalizing at different temperatures in the α + β phase zone. (**a**) 930 °C /2 h/AC, (**b**) enlarged view of the box in Figure 7a, (**c**) 990 °C/2 h/AC, (**d**) enlarged view of the box in Figure 7c.

Figure 7a,b show the SEM photographs at the normalizing of 930 °C/2 h/AC. Figure 7b shows the further enlarged microstructure in the white wireframe displayed in Figure 7a. The lath-shaped mirostructure is a primary α-Ti phase (the gray area as shown in Figure 7a), and the microstructure between the primary α-Ti phases is β-Ti phase or α+β phase (the gray-white area as shown in Figure 7a). There is a white mesh microstructure inside the primary α-Ti phase, which is preliminarily inferred to be β-Ti phase. The original β-Ti phase between the primary α-Ti phases will grow up during the heating and heat preservation process. Also, some of primary α-Ti phases are converted into β-Ti phases because the heating temperature is 930 °C and located in the temperature interval below the α+β two-phase area. Similarly, the β-Ti phase can also be precipitated inside the primary α-Ti lath phase. In the subsequent cooling process, a secondary α-Ti phase is precipitated in the larger β-Ti phase between the primary α-Ti phases, which is the α+β two-phase coexistence area. Due to the non-equilibrium crystallization, the β-Ti phase transforms incompletely and remains between the lath-shaped primary α-Ti phases and presents a elongated shape. Under high temperature and heat preservation conditions the β-Ti phase precipitated in the primary α-Ti phase will remain in the subsequent cooling process and distribute inside the primary α-Ti phase in a white mesh pattern due to the phase transition in the non-equilibrium state.

At normalizing conditions of 990 °C/2 h/AC SEM photographs are shown in Figure 7c,d. Figure 7d is a further enlarged microstructure to the white wireframe in Figure 7c. The microstructure between the primary α-Ti phases transforms into the β-Ti phase completely in high temperature (990 °C) and heat preservation process. At the same time, β-Ti phase is precipitated within the primary α-Ti phase. In the subsequent cooling process, the secondary α-Ti phase (needle-shaped or short lath-shaped) will be precipitated in the subboundary β-Ti phase. The residual subboundary β-Ti phase exists at room temperature due to the phase transition in the non-equilibrium state. Therefore, the microstructure between the primary α-Ti phases at room temperature is α + β two-phase coexistence. The β-Ti phase precipitated inside the primary α-Ti phase is the same as the above analysis result distributed in the lath-shaped primary α-Ti phase in a coarse mesh pattern.

### 3.2. Phase Composition

**Figure 8:**
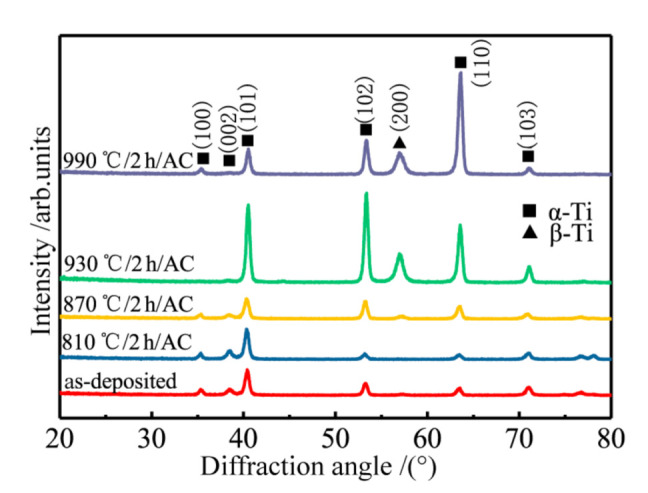
XRD patterns of LDF TC4 titanium alloy under different heat treatments.

The XRD analysis results of TC4 as-deposited and normalized specimens at different temperatures are shown in Figure 8. A large number of diffraction peaks of α-Ti phase exist in the specimens as-deposited and normalized at 840 °C and 870 °C, including (100)_α_, (002)_α_, (101)_α_, (102)_α_, (110)_α_, and (103)_α_, respectively. With the increasing of normalizing temperature, the diffraction peaks intensity of β-Ti phase does not change much. The reason of weak diffraction peaks of the β-Ti phase is that the normalizing temperature in the single phase region of α-Ti phase has not got into the α + β phase region. Therefore, there is no transition from α-Ti phase to β-Ti phase, while the residual β-Ti phase will transform into α-Ti phase increasing and keep the temperature in this region. When the normalizing temperature is 930 °C, the diffraction peaks of (100)_α_ and (002)_α_ disappear. But the diffraction peaks of (101)_α_, (102)_α_, (110)_α_, and (103)_α_ have an obvious increased intensity. The diffraction peak of (200)_β_ appears at 2θ ≈ 57°. At the normalizing temperature of 990 °C the diffraction peak of (101)_α_ and (102)_α_ decreases. On the contrary, the diffraction peak (110)_α_ increases. At the normalizing temperature of 990 °C, the diffraction peak of (200)_β_ is basically the same as that of 930 °C.

### 3.3. Mechanical Property

**Figure 9:**
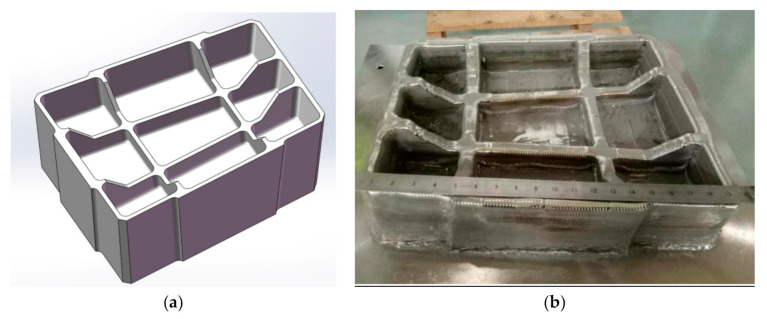
Hanging frame model and physical diagram. (**a**) Model diagram; (**b**) physical diagram.

A frame part with the size of 570 mm × 470 mm × 210 mm is fabricated according to the parameters in Table 1. As the model and physical drawing shown in Figure 9a,b, there are no obvious defects in the surface of workpiece after selective laser melting. Metallographic samples are prepared by cutting the sample using wire electrical discharge machining (WEDM) for testing specimens microstructures and mechanical properties.

**Figure 10:**
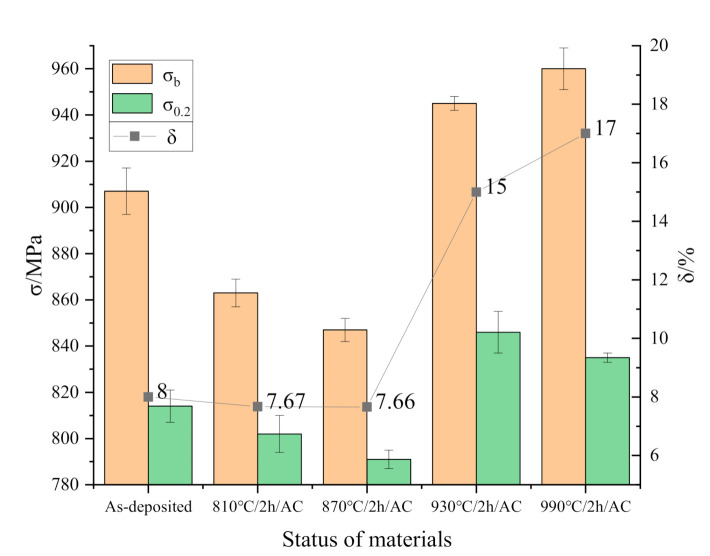
Room temperature tensile properties of Ti-6Al-4V (TC4) alloy by selective laser melting.

The tensile properties of as-deposited specimens and the other four normalizing parameters are chosen to compare in Figure 10. The stress and elongation of each sample were measured three times respectively and the average value was obtained At the normalizing temperatures of 810 °C and 870 °C, the tensile strength (*σ*_b_), yield strength (*σ*_0.2_), and elongation (*δ*) are almost the same as that of the as-deposited state. With the length and width of the α-Ti phase increasing during normalizing treatment, a large number of slender primary α-Ti phases are easy to break and hinder the movement of dislocations resulting in a decrease in the deformation compatibility and strength and plasticity of the specimens. Tensile strength, yield strength and elongation are higher than those of as-deposited phase at normalizing temperature of 930 °C and 990 °C. The elongated primary α-Ti phase will be truncated during the precipitation of β-Ti phase and the primary α-Ti phase will be transformed into short rod-like or equiaxed crystal due to the precipitation of β-Ti phase from primary α-Ti phase at high temperature. The comprehensive mechanical properties of the specimens after normalizing are better than the national standard of forgings (*σ*_b_ ≥ 895 MPa, *σ*_0.2_ ≥ 830 MPa, δ ≥ 10%).

**Figure 11:**
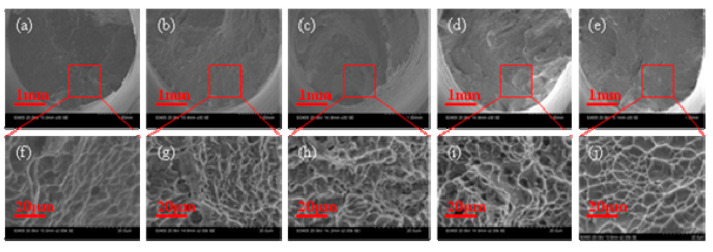
Fractographs of selective laser melting TC4 titanium alloy of tensile test at room temperature. (**a**) As-deposited before normalizing (30X), (**b**) 810 °C/2 h/AC(30X), (**c**) 870 °C/2 h/AC, (30X) (**d**) 930 °C /2 h/AC(30X), (**e**) 990 °C/2 h/AC(30X), (**f**) As-deposited before normalizing (2kX), (**g**) 810 °C/2 h/AC (2kX), (**h**) 870 °C/2 h/AC (2kX), (**i**) 930 °C /2 h/AC (2kX), (**j**) 990 °C/2 h/AC (2kX).

The fracture morphology of the tensile specimens treated at different normalizing temperatures are shown in Figure 11. The large number of dimples in the cross section indicates a ductile fracture. The specimens at the normalizing temperature of 930 °C and 990 °C presents more uniform fracture morphology than that of normalizing temperature 810 °C and 870 °C in the single-phase region of α-Ti.

**Figure 12:**
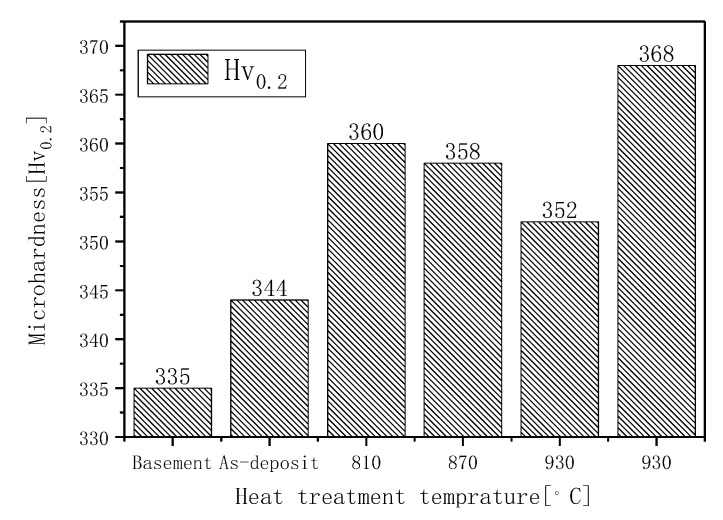
Relational curve of hardness and normalizing temperature.

The microhardness of matrix, as-deposited samples and different normalizing temperatures are shown in Table 4. Above mentioned microstructures evolution trends are confirmed by image analysis data displayed in Figure 12 for the different normalizing temperature by selective laser melting. With the rising of normalizing temperature, the microhardness declines slightly due to coarsening of microstructures. From Figure 12 when the temperature is below 960 °C, the curve of hardness is basically unchanged. While the normalizing temperature is 990 °C, the hardness increases obviously at this time. At this time α phase in the tissue becomes elongated needle shape. This is because the specimen is completely transformed into β at 990 °C. Because of the high temperature and the large temperature gradient with room temperature, even in air cooling, the cooling speed is very fast that makes the transformation of β→α incomplete and the formation of very fine α phase in the cooling process leading to the obvious increase of hardness. The hardness of TC4 titanium alloy made by laser additive manufacturing is high, which is determined by the typical Widmanstätten structure in the deposition area of titanium alloy. The thin lamellar phase and the very fine Widmanstätten lath will increase the hardness in the sedimentary area. On the other hand, rapid heating and cooling increase the solid solubility of the internal phase so as to improve the hardness of titanium alloy deposition area in the process of selective laser melting.

## 4. Conclusions

1. Nearby the fusion line the optical microstructures of the as-deposited samples are coarse columnar crystals of β-Ti phase while there are the optical microstructures of equiaxed crystals at the top. The sub-microstructure in the grains is composed of lath-like primary α-Ti phase and lath-like primary α-Ti phase.
2. When normalizing in the single-phase region of α-Ti phase, the length and width of primary α-Ti phase increase. While the length–width ratio and content of primary α-Ti phase gradually decrease with the increase of normalizing temperature. When normalizing in the two-phase region of α + β, the elongated primary α-Ti phase is truncated by the precipitated β-Ti phase and becomes short rod-like or equiaxed α-Ti phase. The volume fraction of primary α-Ti phase decreases as the length and width of primary α-Ti phase increase. In the single-phase region of α-Ti (810 °C and 870 °C), the β-Ti phase mainly exists between the lath-like primary α-Ti phase. When normalized at 930 °C and 990 °C, the β-Ti phase not only exists between the lath-like primary α-Ti phase, but also precipitates in a network of the lath-like primary α-Ti phase.
3. The normalizing temperature has a significant effect on the room temperature tensile and hardness properties of laser additive manufacturing TC4. When the normalizing condition is at 990 °C/2 h/AC, the best tensile properties matching can be obtained. The tensile strength, yield strength and elongation of TC4 are 960 MPa, 835 MPa and 17% respectively, which are better than the requirements of the national standard of forgings (σb ≥ 895 MPa, σ0.2 ≥ 830 MPa, δ ≥ 10%).

## Figures and Tables

**Table 1:** Laser melting deposition parameters used in this study.

Parameters	Symbol	Unit	Value
Laser beam power	P	W	2200
Laser beam diameter	D	mm	3
Scanning speed	S	mm·min^−1^	800
Lapping rate	H	-	50%
Powder feeding speed	F	g·min^−1^	6.37

**Table 2:** Chemical composition of as-built TC4 material.

Elements	Al	V	Fe	C	O	N	H	Ti
Content (wt%)	5.5–6.75	3.5–4.5	<0.25	<0.08	0.12–0.16	<0.01	<0.01	others

**Table 3:** Normalizing treatments: Lists of parameters.

Temperature/°C	Holding Time/h	Cooling
810 °C	2 h	AC
870 °C	2 h	AC
930 °C	2 h	AC
990 °C	2 h	AC

**Table 4:** Hardness of different normalizing temperature.

Normalizing (°C)	Hardness Test1 (HV0.2)	Hardness Test2 (HV0.2)	Hardness Test3 (HV0.2)	Average (HV0.2)
Basement	332	332	341	335
As-deposited	351	338	343	344
810	357	359	364	360
870	353	357	364	358
930	358	351	347	352
990	366	361	377	368
